# Study on the variable side - deep fertilization control system for rice based on soil electrical conductivity

**DOI:** 10.3389/fpls.2026.1748101

**Published:** 2026-01-26

**Authors:** Lantian Xie, Wenjie Mao, Xincheng Zhang, Yagang Du, Xin Fang, Cheng Zhou

**Affiliations:** 1 School of Information Engineering, Huzhou University, Huzhou, China; 2 Informatics Department, Jingning She Autonomous County People’s Hospital, Lishui, China; 3 Institute of Crop Science, Huzhou Academy of Agricultural Sciences, Huzhou, China; 4 Institute of Agricultural Engineering, Heilongjiang Academy of Land Reclamation Sciences, Harbin, China

**Keywords:** current–voltage method, electrical conductivity, neural network, precision agriculture, rice, variable fertilization

## Abstract

To address the problem that current rice fertilization devices rely on experience-based settings of fertilizer application rates and are unable to dynamically adjust according to soil fertility, resulting in low fertilizer use efficiency, a rice side-deep variable-rate fertilization control system based on real-time soil electrical conductivity (EC) detection was designed. First, a three-factor, four-level full factorial experiment was conducted to investigate the effects of soil moisture content, electrode insertion depth, and soil temperature on soil EC, and an EC calibration model was established based on an RBF neural network. Second, a fertilization strategy was developed and a fertilizer application model was constructed based on real-time EC, target yield, and implement forward speed; meanwhile, an incremental PID algorithm was adopted to achieve closed-loop control of variable-rate fertilization with motor speed as the control objective, completing the control system design. Finally, the system was deployed on a pneumatic groove-wheel fertilizer metering device and field experiments were carried out. The performance of the soil EC detection system and the variable-rate fertilization system was validated through EC detection accuracy tests and variable-rate fertilization system response tests. The results showed that the average relative error of EC was 2.70%, the maximum coefficient of variation of the fertilization system response stability was 3.98%, the maximum response time was 1.60 s and the average was 1.28 s, and the average fertilizer reduction rate was 12.39%. These results indicate that the proposed soil EC detection and variable-rate fertilization system can achieve rapid and accurate variable-rate fertilization operations. This study can provide equipment and technical support for rice side-deep variable-rate fertilization.

## 1 Introduction

As a major rice-producing country (Zhou et al., 2025), China has traditionally adopted fixed-rate fertilization in paddy fields, where application rates are largely determined by experience. This practice is prone to over-fertilization, leading to low fertilizer use efficiency. Taking nitrogen fertilizer—the most commonly used type—as an example, the utilization rate in developed countries is approximately 50%–60%, whereas that in China is about 30%–50%, which is markedly lower than in developed countries (Ren et al., 2023). Excessive fertilizer application not only diminishes the marginal yield gains of grain production and increases input costs, but also imposes adverse impacts on water resources, soil quality, and the environment (Xing and Wang, 2024). Variable-rate fertilization (VRF) systems are a key component of precision agriculture and can effectively reduce fertilizer waste while improving the economic performance of agricultural production (Vergara-Díaz et al., 2016; Wang et al., 2024; Cai et al., 2025). In general, a VRF system consists of a sensing (detection) subsystem and a fertilization (application) subsystem: the sensing subsystem acquires in-field physicochemical information to support decision-making and guide the application subsystem to execute corresponding operations. Common approaches for acquiring such in-field information include soil EC sensing, prescription map generation, and crop growth status monitoring.

Soil EC is a key parameter characterizing the soil environment (Scudiero et al., 2025). It directly reflects the salt content in the soil (Kim and Park, 2024) and is influenced by various physicochemical properties of the soil (Yu and Kurtural, 2020; Benke et al., 2020; Xu et al., 2016). In recent years, soil EC has been increasingly applied in the detection subsystems of variable fertilization systems as an effective indicator for assessing soil fertility and productivity due to its economic feasibility and practicality (Stadler et al., 2015; Gao et al., 2021). In Western countries, soil EC detection has been primarily applied to expand the spatial coverage of detection systems. For example, online EC mapping devices such as the Veris system (USA) and DUALEM (Canada) (Serrano et al., 2014) can collect large-scale soil EC data. However, these systems are bulky and expensive, making them unsuitable for small or medium-sized farmlands or complex terrains. In Japan, Iseki Co., Ltd. developed a rice side-deep fertilization machine (Morimoto et al., 2013) equipped with an onboard soil EC detection system to evaluate soil fertility and guide subsequent fertilization operations. However, its data fusion and decision-making algorithms are relatively simple, and the detection accuracy remains limited. In China, research has mainly focused on improving the accuracy of soil EC detection. Han Changjie (Han et al., 2022) designed a vehicle-mounted rapid soil EC detection and data acquisition system and established a regression model for calculating the leachate EC. However, the system was tested under dryland conditions, where EC measurements were easily affected by soil moisture fluctuations, leading to reduced detection accuracy and model stability. At present, most high-precision soil EC detection systems are designed as independent units, lacking integrated studies that combine high-accuracy EC detection with variable fertilization control systems.

Prescription-map–based approaches generally provide high accuracy for guiding fertilization, and Western countries have established relatively mature workflows for prescription-map generation using remote sensing and historical field-operation data, enabling field zoning and automated variable-rate control. A hydraulically driven variable-rate fertilization system developed by AMAZONE (Germany) based on prescription maps (Chen et al., 2017) has demonstrated good application performance and can significantly increase crop yield; however, it is associated with high costs and considerable operational complexity. In China, Zhang et al. (2021) implemented demand-driven variable fertilization according to soil fertility using prescription-map information and achieved proportioned application of single-nutrient fertilizers, with an application accuracy exceeding 97%. Nevertheless, soil sampling and laboratory measurements are required prior to fertilization, resulting in limited operational flexibility. In general, prescription-map generation relies on technically demanding procedures with high production cost and stringent operational requirements. Moreover, because prescription maps are prepared before fertilization, they typically need to be regenerated for each field and each fertilization event, which further constrains real-time applicability and operational flexibility in field conditions.

Crop growth information–based detection systems rely on canopy reflectance characteristics to guide fertilization operations, primarily using spectral and leaf-surface imaging technologies. Dammer et al. (2008) in Germany developed a variable sprayer based on the leaf area index (LAI) of cereal crops. The system employs a portable spectrometer to monitor crop growth density in real time and integrates GPS data with a hydraulic control valve to achieve automatic spray regulation. However, due to spatial heterogeneity in crop density, when the variation scale is smaller than the boom section width, the system cannot accurately respond to local differences, potentially leading to under-application in high-density zones and over-application in low-density zones. In China, Shi Yinyan et al. (Shi et al., 2018) developed a centrifugal variable-rate fertilization machine for rice, which uses a GreenSeeker spectral system to obtain real-time NDVI values of rice canopies for precise fertilization control. Nevertheless, the NDVI of the canopy only reflects information from the upper leaves, making it difficult to monitor the conditions of lower plant layers. Furthermore, machine-vision-based fertilization systems are easily affected by environmental factors such as water surface reflection and leaf occlusion in paddy fields, which reduces decision-making accuracy.

In recent years, given that soil EC is jointly affected by multiple factors such as moisture content, temperature, and electrode insertion depth, and exhibits highly nonlinear relationships, studies have begun to introduce neural-network models for EC calibration and inversion. Compared with conventional linear or polynomial regression methods, neural networks can better capture the complex mapping between soil physicochemical factors and EC, thereby improving prediction accuracy (Qi et al., 2024) In addition, intelligent optimization methods such as particle swarm optimization (PSO), and genetic algorithms have been combined with RBF networks to optimize the network structure and parameters via global search, and this strategy has been applied in multiple fields with promising performance (Du, 2023).

With the advancement of precision agriculture, a series of improved PID-based algorithms have been proposed, including self-tuning PID, fuzzy PID, and feedforward–feedback integrated PID, to enhance dynamic performance and robustness under operating disturbances and load variations (Wang et al., 2014; Xue et al., 2015). Meanwhile, considering that agricultural equipment is typically controlled by embedded controllers and operates in complex field environments with limited maintenance conditions, many engineering applications still favor PID control and its incremental form due to their relatively simple structure, clear physical interpretability of parameters, and ease of tuning and maintenance (Dong et al., 2025).

Therefore, this study aims to improve the accuracy of soil EC detection and proposes a rice side-deep variable fertilization control system based on soil EC measurement. The system investigates the response of soil EC measured by the current–voltage method under different influencing factors and establishes an RBF neural network regression model for soil EC prediction. Combined with the target yield and corrected EC values, a fertilizer application model is constructed. Based on the machine’s forward speed and an incremental PID control algorithm, the motor speed is adjusted to achieve closed-loop control of variable fertilization. This study integrates a high-precision soil EC detection device with a variable fertilization system, providing both technical and hardware support for rice side-deep variable fertilization.

## 2 Study on soil EC detection system

To establish a calibration model for the soil EC detection system, soil samples were collected from the Balidian Town area in Huzhou City, and the response patterns of soil EC under different influencing factors were investigated. The experimental data were analyzed using Origin Pro software through analysis of variance (ANOVA) to evaluate the significance of each factor and to plot response surface diagrams revealing the interaction effects among factors. On this basis, an RBF neural network-based soil EC calibration model was developed to provide accurate and reliable data support for the variable fertilization system.

### 2.1 Principle of soil EC_a_ detection

Common soil apparent electrical conductivity(EC_a_) detection methods mainly include the current-voltage method (Mazur et al., 2022), soil extract method (Kremer et al., 2021, time domain reflectometry (TDR) (Lian, 2009), and electromagnetic induction method (Wang et al., 2025). Since the primary working environment of this detection system is paddy fields, where the field conditions are complex, the EC detection system must not interfere with the operation of the rice transplanter, and its installation position is limited to a single wheel. The current–voltage method features high sensitivity and strong integration capability; therefore, this system adopts a soil EC detection approach based on the current-voltage method.

**Figure 1:**
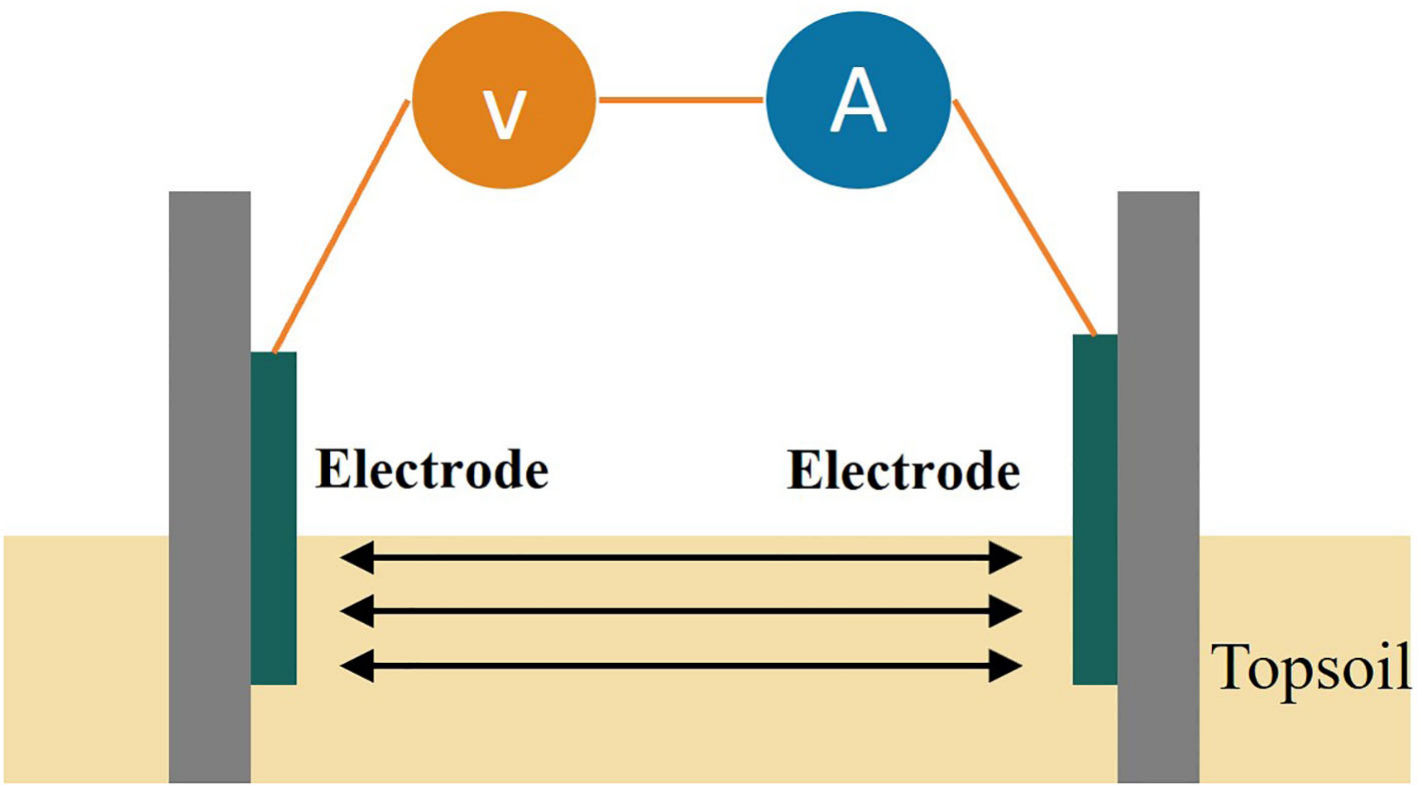
Schematic diagram of conductivity test structure.

In the current-voltage-based soil EC_a_ measurement, a two-electrode electrical conductivity sensor (ECS) applies a constant excitation signal to a pair of electrodes. Variations in the EC of the soil medium cause corresponding changes in the current flowing through the electrode pair. By using conductivity instead of resistivity, and conductance instead of inter-electrode resistance, the EC_a_ of the soil medium can be accurately measured, as shown in **Figure 1**.

The EC ∂ between the electrodes is the reciprocal of the resistance R. Thus, ∂ is calculated according to Equation 1.

(1) $$\begin{array}{c} \partial =\frac{1}{R}=\frac{I}{U} \end{array}$$

Where R is the soil resistance; U is the voltage; I is the current.

The current-voltage method has the advantages of being structurally simple and reliable, but the measurement error of EC_a_ is relatively large. Soil EC is influenced by soil temperature, soil moisture content, and the depth of electrode insertion. Therefore, it is necessary to investigate the effects of these factors on EC in order to maintain a simple detection structure while improving measurement accuracy.

### 2.2 Study on the response patterns of soil EC to different factors

To explore the response patterns of soil EC under different influencing factors, it is necessary to identify the main factors affecting EC measurement. Previous studies have shown that soil temperature, soil moisture content, and electrode insertion depth are all correlated with EC (Corwin and Lesch, 2005; Seo et al., 2022).

Hence, this study selects these three factors to investigate their effects on EC. According to the characteristics of paddy field soil during the irrigation period, soil moisture content is maintained between 70% and 80% (Deshabandu et al., 2024). Therefore, four levels of soil moisture content were selected: 70%, 75%, 80%, and 85%. When the electrode insertion depth in the soil layer is within 0–100 mm, the sensor output voltage is proportional to the insertion depth. When the depth exceeds 100 mm, the sensor output voltage remains nearly constant (Liang et al., 2022). Accordingly, four levels of electrode insertion depth were selected: 40 mm, 70 mm, 100 mm, and 130 mm. Studies also indicate that an increase in temperature enhances the ion diffusion coefficient and thus the conductivity (Zhao et al., 2025). Considering the environmental temperature variation during rice transplanting, four levels of soil temperature were selected: 15°C, 20°C, 25°C, and 30°C. The experimental factor levels are listed in **Table 1**.

**Table 1:** Levels of experimental factors.

Level	Soil moisture content(%)	Soil temperature (°C)	Electrode insertion depth (mm)
1	70	15	40
2	75	20	70
3	80	25	100
4	85	30	130

To eliminate the interference of variations in soil moisture content on the experimental results, soil samples were collected from Balidian Town, Huzhou City, and dried in a 105°C constant-temperature oven for 24 h. The dried samples were then ground and sieved to ensure uniform moisture distribution in the experimental materials.

To guarantee the accuracy of the experimental factor levels, the experiments were conducted in late November 2024 at the Key Laboratory of Agricultural Resources Intelligent Management and Application, Huzhou Normal University. Samples were prepared using water spraying with plastic film sealing and stored in a constant-temperature oven. Each individual soil sample was sealed according to its classification, and the indoor temperature was maintained at 22°C.

According to the experimental design requirements, the soil surface was sprayed with water and left to stabilize under a plastic cover. Soil moisture content was measured using an FK-QX08 meteorological environment detector. If the moisture content did not reach the target experimental level, the spray-settle-measure cycle was repeated.

To ensure the physical consistency between the sample soil and field soil, the pretreated soil samples were weighed and placed into 5 L beakers. The bulk density of the soil for each trial was maintained at 1.2-1.3 g/cm³. The bulk density ( $\rho_{b}$) was calculated using Equation 2.

(2) $$\begin{array}{c} \rho_{b}=\frac{m}{v} \end{array}$$

where $\rho_{b}$ is the bulk density of the soil, m is the mass of the soil in the beaker, and v is the volume of the soil in the beaker.

After confirming the consistency of soil bulk density, the beakers were sealed with plastic film and placed in a constant-temperature oven for 1 h. Subsequently, the temperature levels of the soil samples were checked to ensure stability, completing the preparation of the soil samples. EC_a_ measurement was then carried out. According to the experimental levels in **Table 1**, a self-developed paddy field soil ECS was inserted into the corresponding soil samples, and the sensor data were recorded as the measured soil EC_a_ values. Soil samples were collected from each beaker at four electrode insertion depths using a ring knife method, and the soil EC was determined using the soil extract method (Bai et al., 2013), which served as the standard soil EC value. To reduce measurement errors, three points were randomly selected on the same sample for repeated measurements, and the mean value was taken as the experimental measurement result.

Based on the experimental results, a quadratic polynomial response surface model was established. ANOVA was performed to test the significance of the main effects and interaction effects, as shown in **Table 2**. According to the p-values, soil moisture content (A), soil temperature (B), and electrode insertion depth (C), all had highly significant effects on soil EC (P< 0.01). Based on the F-values, the order of influence of the three factors on soil EC was electrode insertion depth > soil moisture content > soil temperature. Among these, electrode insertion depth had the most significant impact on measurement accuracy, and a reasonable insertion depth could effectively improve measurement stability. Moisture content played a dominant role in EC variation and was a key factor affecting soil ion mobility. Although temperature was a secondary factor, it still significantly promoted ion migration rate and electrolyte activity.

**Table 2:** Significance analysis of the regression model of electrical conductivity.

Sources of error	Square of sum	Degrees of freedom	Mean square	F value	P value
Model	65285.41	9	7253.93	111.00	< 0.0001
A- Soil Moisture Content	21700.05	1	21700.05	332.06	< 0.0001
B- Soil Temperature	9926.45	1	9926.45	151.90	< 0.0001
C- Electrode Insertion Depth	31285.83	1	31285.83	478.75	< 0.0001
A^2^	144.02	1	144.02	2.20	0.1812
B^2^	333.01	1	333.01	5.10	0.0586
C^2^	1495.67	1	1495.67	22.89	0.0020
Residual	457.44	7	65.35		
Lack of Fit	323.55	3	107.85	3.22	0.1440
Pure Error	133.90	4	33.47		
Corrected Total	65742.85	16			

**Figure 2:**
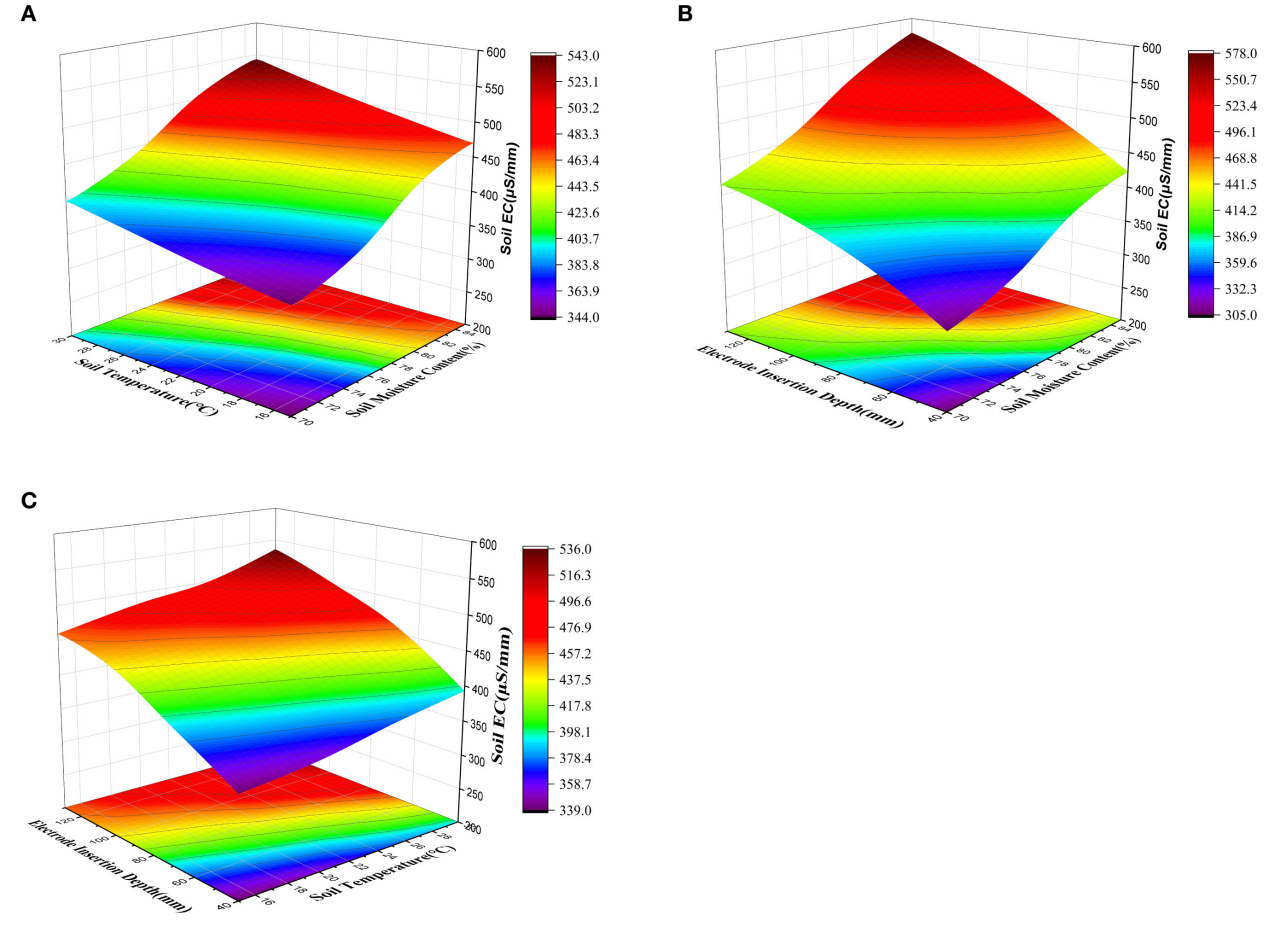
Response surface of various factors affecting EC: **(A)** Response surface of soil moisture content and soil temperature; **(B)** Response surface of soil moisture content and electrode insertion depth. **(C)** Response surface of soil temperature and electrode insertion depth.

Response surface plots showing the interaction effects of these factors on soil EC were drawn using Origin Pro, as illustrated in **Figure 2**. The slope of the response surface reflects the degree of influence of each factor on EC—the steeper the slope, the greater the impact.

On this basis, to overcome the limitations of traditional response surface models in fitting complex nonlinear relationships, an RBF neural network algorithm was introduced to establish a soil EC calibration model. The data obtained from preliminary experiments were used as training samples, and network optimization algorithms were applied to adjust model parameters, achieving high-precision EC Prediction. This method combines the interpretability of statistical analysis with the nonlinear fitting capability of neural networks, providing both theoretical and data support for soil EC prediction.

### 2.3 Construction of the PSO–RBF soil EC calibration model

To minimize the interference of uncontrollable environmental factors, a radial basis function (RBF) neural network—known for its strong capability in handling nonlinear relationships—was employed to construct a soil EC calibration model. The coefficient of determination (R²) and the root mean square error (RMSE) were used as the performance evaluation metrics for the model.

A three-layer RBF neural network (input layer, hidden layer, and output layer) was established to develop the soil EC calibration model based on the current–voltage method. Soil temperature, electrode insertion depth, soil moisture content, and soil EC_a_ were selected as the four input variables, resulting in four neurons in the input layer. The output layer contained one neuron, representing the optimized soil EC. After repeated testing, the number of neurons in the hidden layer was determined to be eight.

To further enhance the model’s global optimization capability, the particle swarm optimization (PSO) algorithm was introduced to optimize the parameters of the RBF network. The core concept of PSO is inspired by the cooperative foraging behavior of bird flocks (Qiu et al., 2024). Each particle carries both velocity and position information, where the position represents a potential optimal solution to the problem. During the search process, particles iteratively update their velocity and position based on their individual best solution and the global best solution of the swarm, as described in Equations 3, 4, thereby achieving a global optimum.

(3) $$\begin{array}{c} V_{i}^{\left(t+1\right)}=WV_{i}^{\left(t\right)}+c_{1}r_{1}\left(P_{ib}-X_{i}^{\left(t\right)}\right)+c_{2}r_{2}\left(g_{b}-X_{i}^{\left(t\right)}\right) \end{array}$$

(4) $$\begin{array}{c} X_{i}^{\left(t+1\right)}=X_{i}^{\left(t\right)}+V_{i}^{\left(t+1\right)} \end{array}$$

Where i is the historical index, t is the number of iterations, W is the inertia weight, $P_{ib}$ is the individual best, $g_{b}$ is the global best, $c_{1}$ and $c_{2}$ are the acceleration coefficients, and $r_{1}$ and $r_{2}$ are random numbers between 0 and 1.

Due to the simplicity of implementation and strong global search capability of the particle swarm algorithm, it effectively avoids the randomness and local optimum problems in selecting the centers, widths, and weight parameters of the traditional RBF network. By globally optimizing network parameters, the PSO-RBF model demonstrates superior performance in nonlinear mapping capability, convergence speed, prediction accuracy, and robustness.

To obtain a PSO configuration that balances predictive performance and computational efficiency, this study performed parameter optimization and sensitivity analysis for the particle number, inertia weight, and maximum iteration count. Based on prior studies and preliminary experiments, the initial search ranges were set to 20–60 particles, an inertia weight of 0.4–0.9, and a maximum iteration count of 20–100. Within these ranges, models were trained under different parameter combinations. RMSE and R² on the validation set were used as the primary evaluation metrics, while convergence speed and computation time were also considered to identify parameter intervals with favorable overall performance.

The coarse-tuning results indicated that when the particle number was below 20 or the iteration count was below 30, the swarm search was insufficient and model accuracy deteriorated markedly. As the particle number and iteration count increased, the validation RMSE decreased gradually; however, when the maximum iteration count approached 50, the reduction in validation error slowed substantially, suggesting that PSO was entering a convergence regime. Considering both accuracy and computational cost, the parameter ranges were further narrowed to 20–40 particles, an inertia weight of 0.4–0.6, and a maximum iteration count of 30–60.

Within the reduced parameter space, sensitivity analysis was conducted for the particle number, inertia weight, and iteration count. The particle number was set to 20, 25, 30, 35, and 40; the inertia weight was set to 0.4, 0.5, and 0.6; and the iteration count was set to 30, 35, 40, 45, and 50.

**Figure 3:**
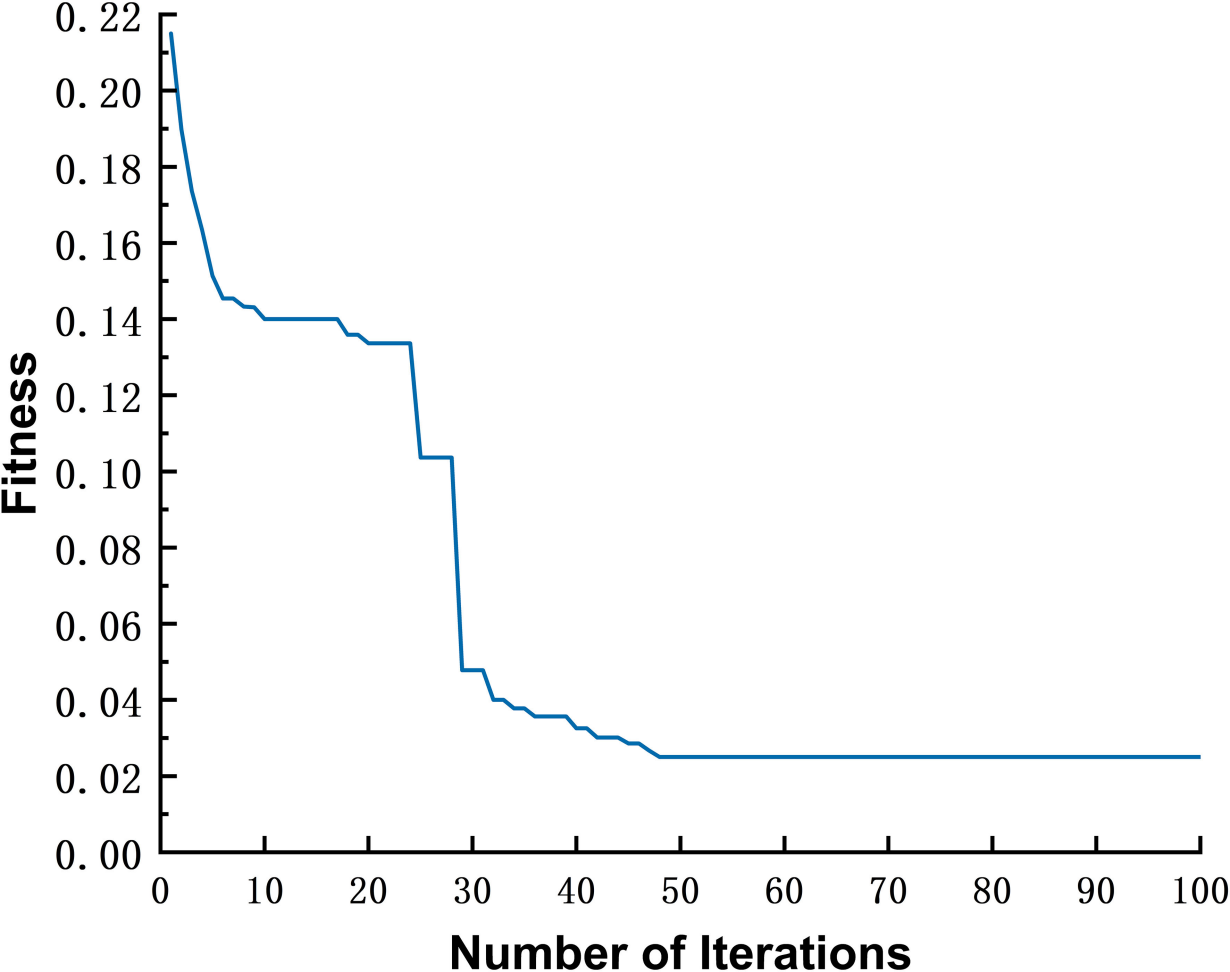
Fitness Evolution Curve during the PSO Algorithm Iteration.

**Figure 3** shows that the PSO algorithm converges after approximately 30–50 iterations, with the optimal number of iterations determined to be 48. According to **Table 3**, when the particle number is 30, inertia weight is 0.5, and the number of iterations is 48, the model performs best on the validation set. Therefore, the final parameters for the PSO-RBF soil conductivity prediction model are selected as a particle number of 30, inertia weight of 0.5, and a maximum iteration count of 48.

**Table 3:** PSO-RBF prediction performance under different PSO parameter settings.

Group number	Number of particles	Inertia weight	Number of iterations	RMSE
1	20	0.4	48	26.58
2	25	0.4	48	22.12
3	30	0.4	48	20.61
4	35	0.4	48	17.87
5	40	0.4	48	19.11
6	20	0.5	48	23.93
7	25	0.5	48	21.55
8	30	0.5	48	10.32
9	35	0.5	48	12.56
10	40	0.5	48	13.11
11	30	0.6	48	24.09

**Figure 4:**
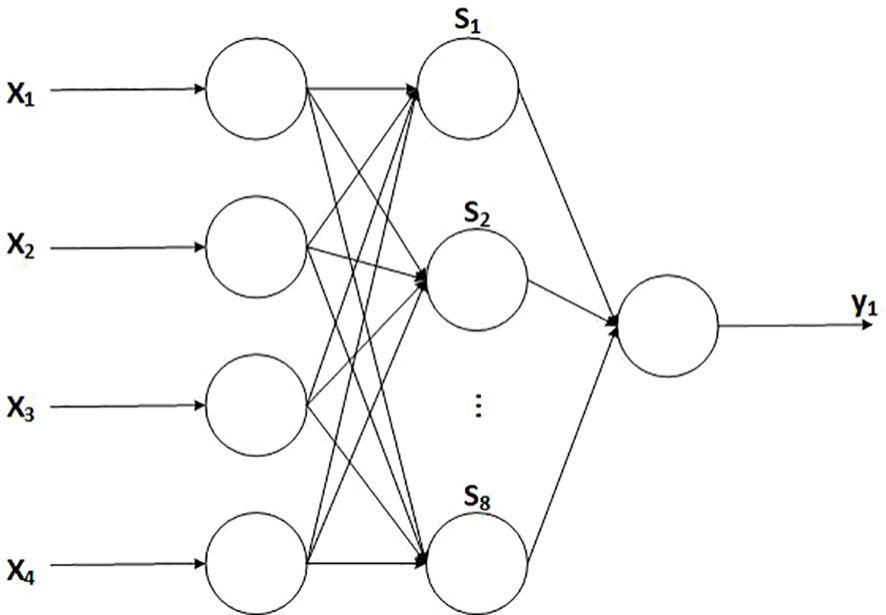
Model structure diagram. x_1_ represents the soil temperature measured by the temperature and humidity sensor; x_2_ represents the soil moisture measured by the temperature and humidity sensor; x_3_ represents the tillage depth measured by the ultrasonic sensor; x_4_ represents the soil EC_a_ measured by the ECS; s_1_–s_8_ represent the hidden layer nodes; y_1_ represents the optimized soil EC.

The PSO-RBF model was trained using Matlab R2023a, with data obtained from the soil EC influencing factor experiment described in Section 2.2. A total of 64 sets of data were used, of which 45 sets were employed for model testing. The optimized RBF neural network model adopts a 4-8–1 structure with normalization in the range [0,1]. When the network output error meets the condition ≤0.000001, the model performance is shown in **Figure 4**, and the model data are presented in **Table 4**.

**Table 4:** Parameter settings for neural network model.

Relevant parameter	RBF model	PSO-RBF model
Training Epochs	1000	1000
Training Target	0.005	0.005
Learning Rate	0.01	0.01
Number of Particles	/	30
Number of Iterations	/	48
Inertia Weight	/	0.5
Search Space Dimension	/	300

To further assess the performance gains brought by PSO-based optimization, data from the sampling experiments that were not used for model training were selected as the test set to evaluate the neural-network models. A no-model condition, defined as using the raw output without applying any calibration model, was adopted as the baseline. **Table 5** presents a comparison of the no-model baseline, the conventional RBF model, and the PSO-RBF model across the evaluation metrics. The results show that the PSO-optimized RBF model outperformed both the conventional RBF model and the no-model baseline in terms of RMSE and R², indicating a substantial improvement in prediction accuracy.

**Table 5:** Results of the model comparison.

Model	RMSE	R^2^
No-model baseline	31.4	0.87
RBF Model	19.1	0.93
PSO-RBF Model	10.32	0.97

### 2.4 Hardware design of the soil EC detection system

**Figure 5:**
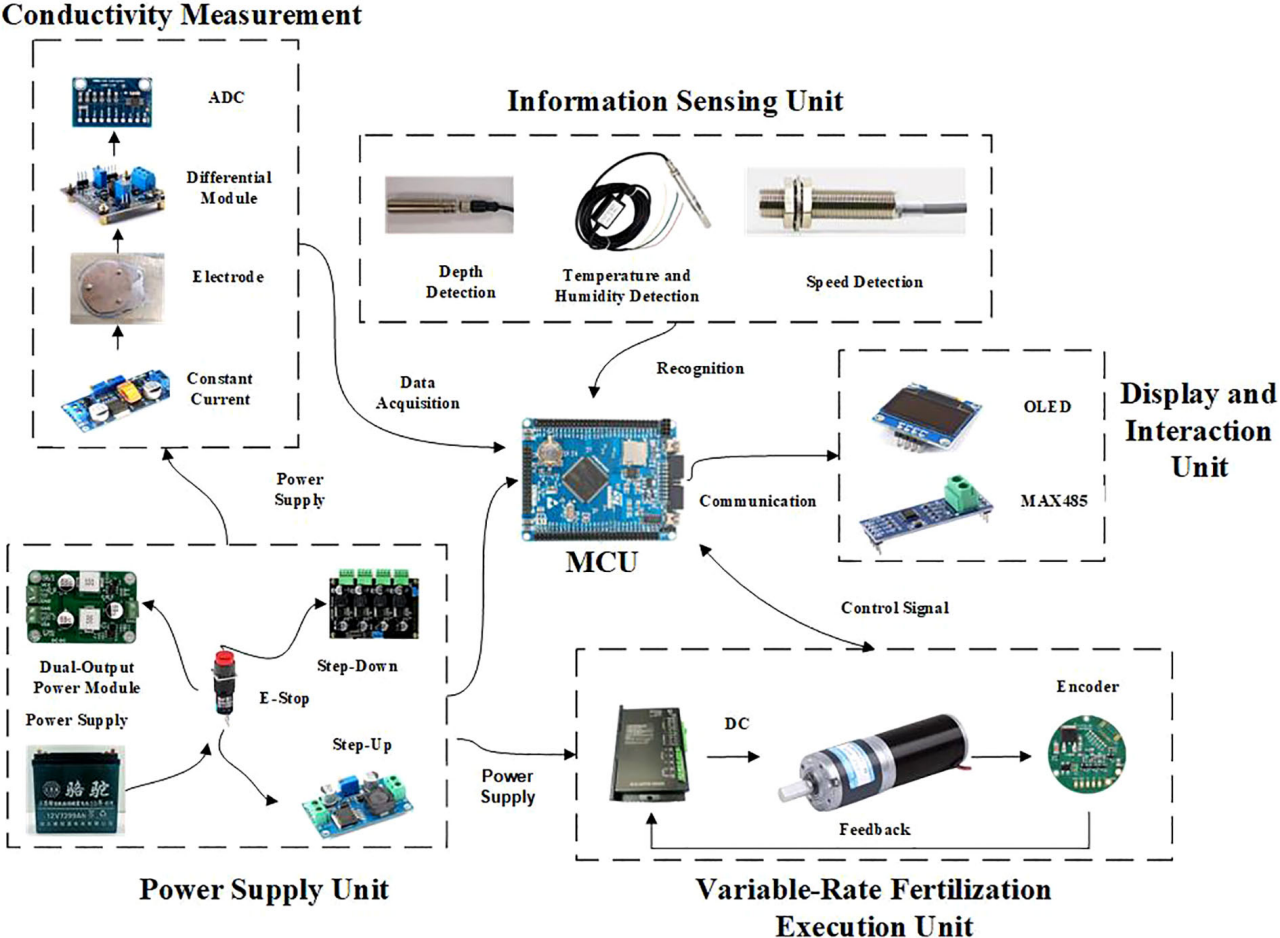
Physical diagram of control system hardware.

**Figure 6:**
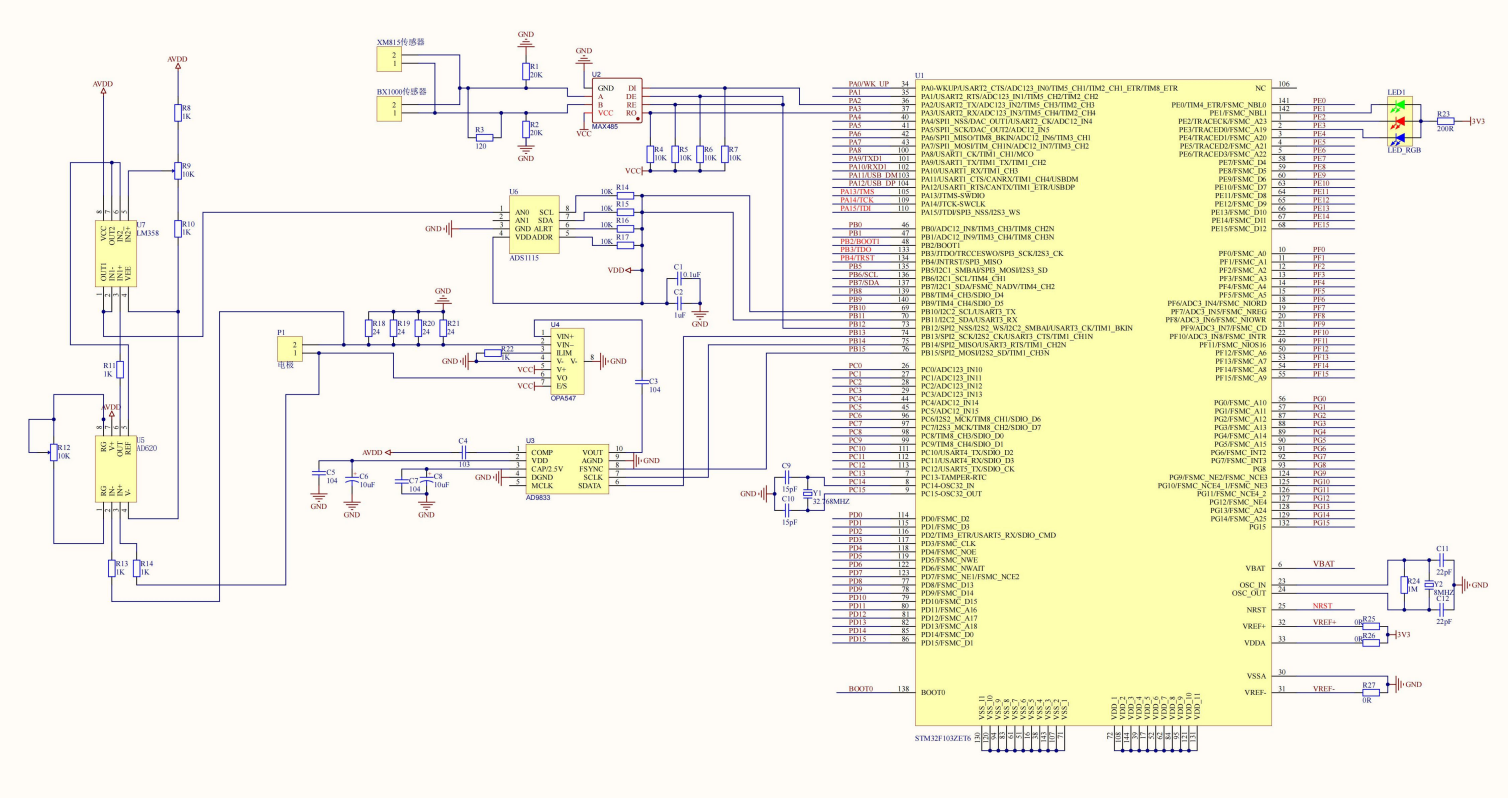
Circuit diagram of EC measurement device.

To meet the system requirements for multi-source sensor data fusion and high-frequency computation of the neural network model, this study adopts a distributed control architecture, dividing the system into two functional modules: the decision control unit and the execution control unit. The decision control unit is responsible for detecting soil EC_a_, performing real-time computations of the calibration model, sensing depth parameters and operating speed, collecting environmental information, generating variable fertilization control commands, and managing human-machine interaction and communication. The execution control unit handles tasks such as driving the fertilizer dispensing mechanism, controlling motor speed, providing status feedback, and interpreting and responding to control commands. The distributed architecture reduces the computational load on the main control chip, thereby improving the overall system response speed and operational efficiency. The control core adopts the STM32F103ZET6 microcontroller based on a 32-bit ARM Cortex-M3 core, which meets the requirements for real-time computation and multitask scheduling. The control system is shown in **Figure 5**, and the circuit schematic is shown in **Figure 6**. The overall hardware structure of the system consists of the power supply unit, EC_a_ detection module, information sensing unit, main control unit, variable fertilization execution unit, and display and interaction unit. The system uses a 12 V lead-acid battery as the main power source, which is regulated by a voltage conversion module to provide stable voltage output for each functional unit.

The EC_a_ detection module is designed based on the current-voltage method and includes a constant-current excitation source, electrodes, and a differential signal acquisition circuit. During field operations, it continuously acquires *in-situ* soil EC_a_ signals, which are then transmitted to the main control unit after differential signal processing. The main control unit synchronously collects tillage depth data from the ultrasonic sensor and soil environmental data from the temperature and humidity sensor. These data are used to dynamically correct the soil EC_a_ value using the calibration model, and variable fertilization control commands are then generated through the fertilization decision model.

## 3 Variable fertilization system design

### 3.1 Structure design of the fertilization system

A Kubota rice transplanter was used as the main body, and a rice transplanting and simultaneous side-deep fertilization integrated machine was improved accordingly. The temperature and humidity sensors and the depth detection sensor in the information perception unit are responsible for real-time monitoring of soil conditions and tillage depth during machine operation. Together with signals from the EC_a_ detection module, these data are provided as inputs to the main control unit. The encoder continuously monitors the fertilizer discharge status and feeds back the information to the fertilizer control system, forming a closed-loop control mechanism. The OLED display module presents key operational parameters in real time, such as operating status, EC values, and fertility indices, enabling operators to monitor the fertilization process conveniently. The system also supports parameter configuration and remote management via a serial communication interface with the host computer, achieving intelligent scheduling of the entire variable fertilization control system.

**Figure 7:**
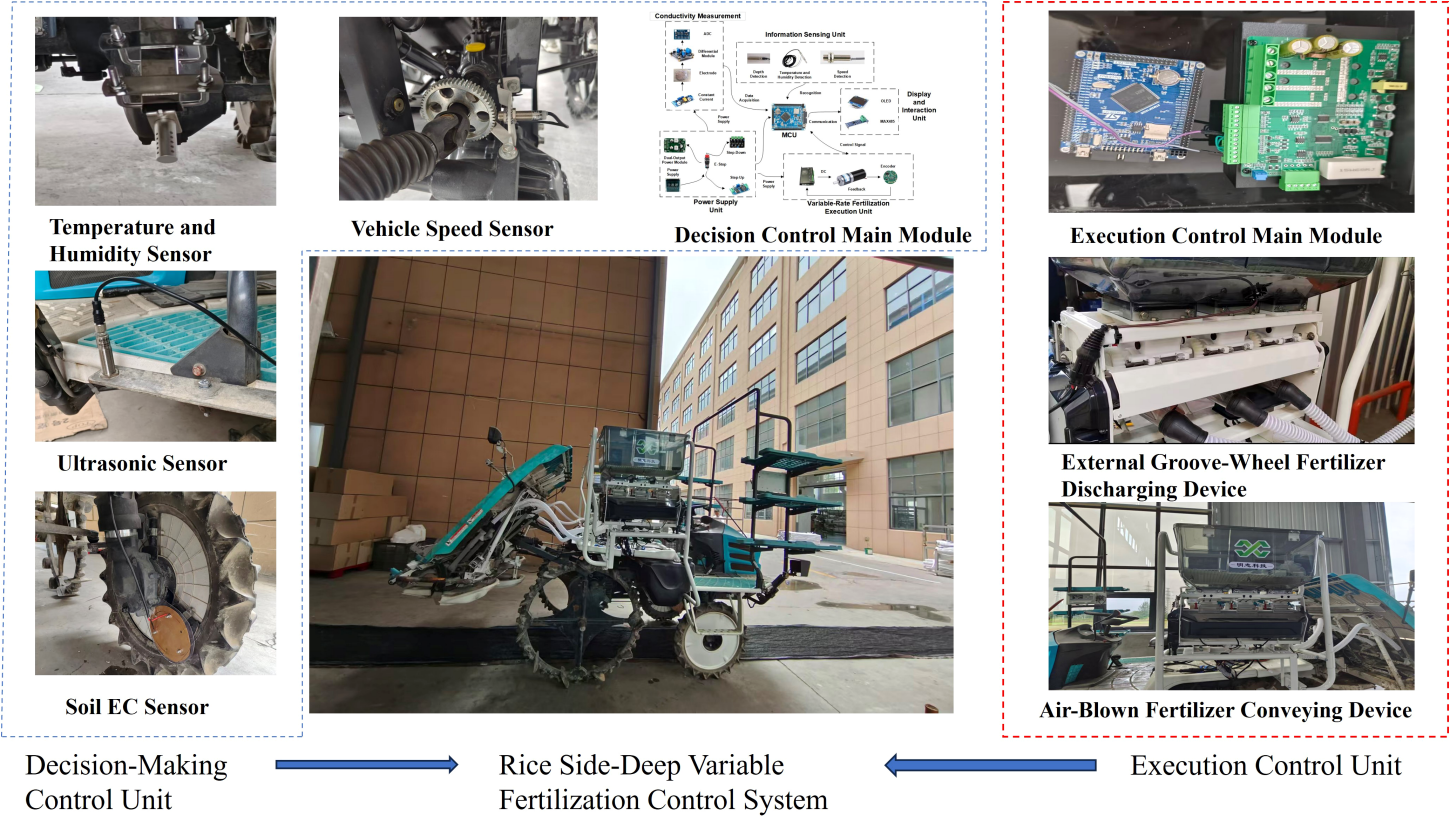
Variable rate fertilization system.

As shown in **Figure 7**, the integrated machine equipped with an EC-based side-deep variable fertilization control system mainly consists of a Kubota riding-type rice transplanter, an EC_a_ detection sensor, temperature and humidity sensors, an ultrasonic sensor, a speed sensor, a control system, a power supply system, an external-groove-wheel variable fertilizer-discharging device, and a pneumatic fertilizer-delivery device. The control system includes three functional modules: an EC_a_ detection subsystem, a decision-making unit, and an execution unit for the variable fertilization system.

The external-groove-wheel fertilizer-discharging device is composed of a fertilizer tank, housing, chain drive–lead screw mechanism, groove-wheel movable baffle, and fertilizer outlet pipe. The pneumatic fertilizer-delivery device consists of a high-power DC centrifugal fan, air delivery pipe, and fertilizer transport pipe. After receiving control commands from the main controller, the execution unit drives the DC motor to adjust the rotational speed of the fertilizer shaft. Through the chain drive-lead screw mechanism, the fertilizer discharge rate is precisely and dynamically controlled. The stability and real-time performance of the control system are key to improving the overall performance of the variable fertilization system. Therefore, it is necessary to establish a fertilization system model and optimize the closed-loop control algorithm.

### 3.2 Establishment of the fertilizer application amount model

The main methods for calculating fertilizer application amount include the nutrient balance method, the nutrient abundance-deficiency index method, and the fertilizer effect function method. Among them, the nutrient balance method estimates the nutrient supply capacity of the soil by measuring its nutrient content and the corresponding basic yield. Compared with the other two methods, the soil nutrient content shows a stronger correlation with soil EC. Therefore, the nutrient balance method was adopted in this study to calculate the fertilizer application amount (Sun, 2024), and the fertilizer application amount was calculated using Equation 5.

(5) $$\begin{array}{c} Q=\frac{V_{N}S\;-\;\left(0.19k\;+\;3.4\right)\mu_{N}}{\omega_{f}\eta_{Nf}} \end{array}$$

where Q represents the fertilizer application amount corresponding to the current soil EC; $V_{N}$ is the nutrient uptake per unit crop yield ( $V_{N}$ = 0.006); S denotes the target yield; k is the current soil EC; $\mu_{N}$ is the correction coefficient of available nutrients ( $\mu_{N}$ = 0.5); $\omega_{f}$ is the nutrient content in the fertilizer ( $\omega_{f}$ = 0.46); and $\eta_{Nf}$ is the fertilizer utilization rate ( $\eta_{Nf}$ = 0.3).

The fertilization mechanism of this system adopts an external groove wheel metering device. A relationship model was established between the rotational speed of the control motor and the fertilization amount (Wang et al., 2023). The target motor speed in the operating area is calculated as follows:

(6) $$\begin{array}{c} Q=\frac{nmq}{10LV} \end{array}$$

where Q is the target fertilization amount, n is the number of fertilizer dispensers, m is the amount of fertilizer discharged per revolution of the dispenser, L is the working width of the machine, and V is the travel speed of the implement.

(7) $$\begin{array}{c} q=q^{\prime }K_{1}K_{2} \end{array}$$

Where $q^{\prime }$ is the rotational speed of the fertilization control motor, $K_{1}$ is the reduction ratio of the planetary gearbox ( $K_{1}$ = 1/19), and $K_{2}$ is the transmission ratio between the output shaft of the gearbox and the external groove-wheel fertilizer shaft ( $K_{2}$ = 1).

By combining Equations 6, 7, we obtain

(8) $$\begin{array}{c} q^{\prime }=\frac{190LVQ}{nm} \end{array}$$

During operation, the environmental data of the working area are collected and, combined with the soil EC Calibration model, are input into the fertilization amount model to obtain the target fertilization amount, Q. By monitoring the operating speed V of the variable-rate fertilization machine in real time and substituting it into Equation 8, the target rotational speed $q^{\prime }$, of the application-control motor can be calculated. The central controller converts the target motor speed into a PWM duty cycle signal to generate the control signal. The PWM signal is then sent to the fertilizer discharge controller to drive the motor. Simultaneously, the encoder monitors the actual rotational speed of the control motor and feeds it back to the central controller. Through closed-loop control of the motor speed, precise regulation of the fertilizer application amount is achieved.

### 3.3 Closed-loop control algorithm of the fertilization control system

The PID control algorithm, as a classical feedback control method (Liu et al., 2024; Huang et al., 2024; Huai et al., 2015; Kamgar et al., 2015; May and Kocabiyik, 2015), has various types to adapt to different system requirements. Incremental PID control offers practical engineering advantages, including a simple structure, ease of implementation, and relatively convenient parameter tuning and maintenance (Jia et al., 2022). In contrast, advanced control methods such as fuzzy control and model predictive control generally provide stronger adaptability under complex operating conditions, but their algorithmic complexity is higher and they impose greater demands on computational resources and implementation conditions, making it difficult to achieve stable, real-time deployment in large-scale agricultural production. Therefore, this study selected incremental PID as the closed-loop control strategy for the actuation motor.

**Figure 8:**
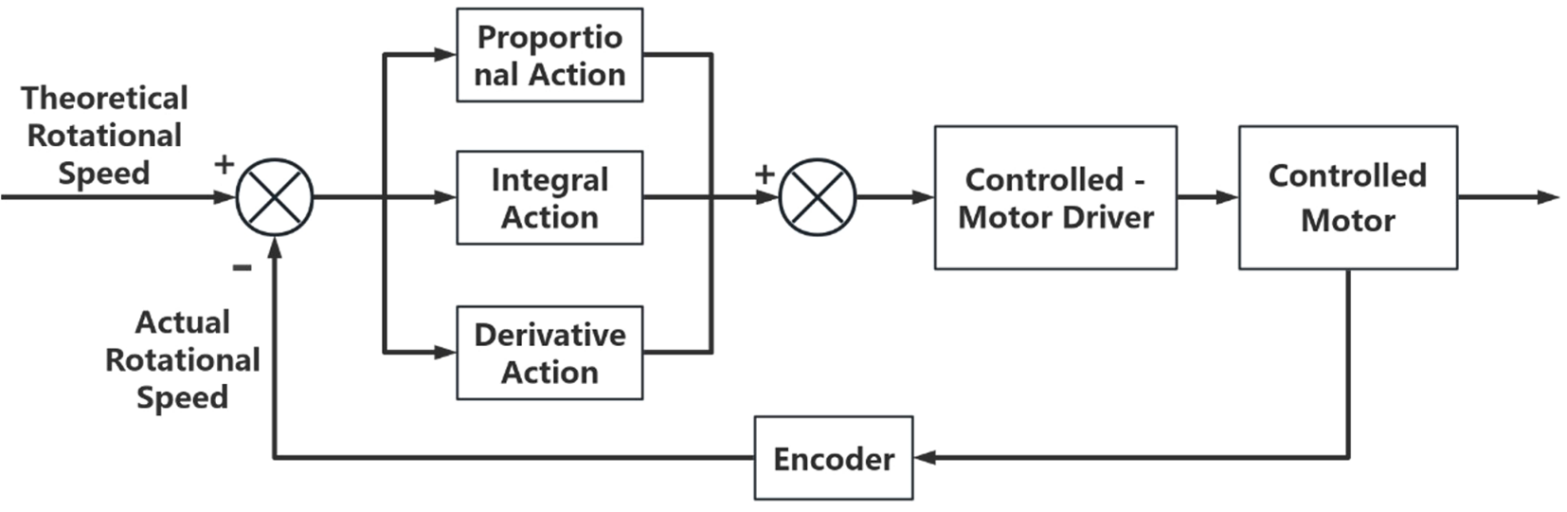
PID system block diagram.

To improve the accuracy of fertilization and the control performance at low travel speeds, a high-precision magnetic encoder was installed at the end of the fertilization control motor. The encoder operates in the central controller’s encoder mode, counting at four times the frequency to monitor the actual rotational speed of the control motor. The actual motor speed information is fed back to the central controller, where the deviation from the theoretical motor speed is calculated. This deviation is processed through proportional, integral, and derivative operations to form a closed-loop control for the fertilization control motor. The control system block diagram is shown in **Figure 8**.

The deviation is expressed as follows (Equation 9):

(9) $$\begin{array}{c} e\left(t\right)=s\left(t\right)-l\left(t\right) \end{array}$$

where e(t) represents the deviation between the actual and theoretical values, s(t) is the actual rotational speed of the fertilization control motor, and l(t) is the theoretical rotational speed of the fertilization control motor. The larger the value of e(t), the greater the difference between the actual operating state of the motor and the desired state.

The traditional PID control algorithm is expressed as follows (Equation 10):

(10) $$\begin{array}{c} u\left(t\right)=K_{P}\left[e\left(t\right)+\frac{1}{T_{I}}\int_{0}^{t}e\left(t\right)dt+T_{D}\frac{de\left(t\right)}{dt}\right] \end{array}$$

where u(t) is the output of the PID controller, $K_{P}$ is the proportional gain, $T_{I}$ is the integral time constant, and $T_{D}$ is the derivative time constant.

The above PID algorithm represents a typical linear control method. However, in computer-based control, the process is essentially a sampled-data control system, and the control action must be calculated based on the deviation at discrete sampling instants. Under this condition, the continuous form of the PID control model is no longer applicable and must be discretized to obtain a discrete PID algorithm suitable for digital control systems, as shown in Equation 11.

(11) $$\begin{array}{c} u\left(k\right)=K_{P}\left[e\left(k\right)+\frac{1}{T_{I}}\sum_{i=0}^{k}e\left(i\right)T+T_{D}\frac{e\left(k\right)-e\left(k-1\right)}{T}\right] \end{array}$$

where T is the sampling period, and k is the sampling sequence, with k = 1, 2…

That is:

(12) $$\begin{array}{c} u\left(k\right)=K_{P}e\left(k\right)+K_{I}\sum_{i=0}^{k}e\left(i\right)+K_{D}\left[e\left(k\right)-e\left(k-1\right)\right] \end{array}$$

where $K_{I}$ is the integral coefficient, and $K_{D}$ is the derivative coefficient.

From Equations 10, 12, it can be seen that the output u(k) of the control model at the k -th sampling instant is related to the outputs at all previous sampling instants. The deviations e(t) from each sampling instant must be cumulatively summed, which increases the computational load of the central controller. Moreover, if the control variable reaches its maximum value, continued accumulation of e(t) under the integral action may easily lead to system instability. To address these shortcomings, the control model is further optimized.

According to the recursive principle, the controller output at the (k−1) -th sampling instant is expressed as:

(13) $$\begin{array}{c} u\left(k-1\right)=K_{P}e\left(k-1\right)+K_{I}\sum_{i=0}^{k-1}e\left(i\right)+K_{D}\left[e\left(k-1\right)-e\left(k-2\right)\right] \end{array}$$

By subtracting Equation 12 from Equation 13, we obtain:

(14) $$\begin{array}{c} \text{Δ}e\left(k\right)=K_{P}\left[e\left(k\right)-e\left(k-1\right)\right]+K_{I}e\left(k\right)+K_{D}\left[e\left(k\right)-2e\left(k-1\right)+e\left(k-2\right)\right] \end{array}$$

As shown in Equation 14, once the three coefficients $K_{P}$, $K_{I}$, and $K_{D}$ of the incremental PID control algorithm are determined, the deviation no longer needs to be cumulatively summed. The controller’s output increment can be calculated using only the deviations from the three most recent sampling instants. This prevents sudden fluctua-tions in the rotational speed of the fertilization control motor due to unexpected events, reduces the computational load of the system, and significantly improves the stability and reliability of the fertilization control system.

After the incremental PID structure is determined, the PID parameters $K_{P}$, $K_{I}$, and $K_{D}$ need to be properly tuned. This study uses the Ziegler–Nichols (ZN) tuning method to obtain the initial PID parameters and then performs engineering fine-tuning based on system performance indices. The specific steps are as follows: first, the integral and derivative terms are set to zero, and only the proportional term is retained. $K_{P}$ is gradually increased. When the system output exhibits sustained oscillations with equal amplitude, the ultimate gain $K_{u}$ and the ultimate oscillation period $T_{u}$ at this moment are recorded, and the initial estimates of the PID parameters are given accordingly. Then, engineering tuning is carried out on the basis of these initial values: the integral term $K_{I}$ is introduced first and gradually adjusted to control the steady-state error within ±3%; next, the derivative term $K_{D}$ is added to suppress overshoot and reduce rapid fluctuations in the actuator motor speed. During tuning, a settling time of less than 2.0 s, an overshoot of less than 5%, and smooth motor-speed variation without obvious oscillation are used as constraint indices, and finally the PID parameter settings that meet the operating requirements of rice side-deep variable fertilization are obtained.

## 4 Results and analysis

### 4.1 EC detection accuracy test

To evaluate the performance of the soil EC calibration model, a field accuracy test was conducted on June 2, 2025, at the Qinglv Smart Farm in Danyang, Zhenjiang, Jiangsu Province.

**Figure 9:**
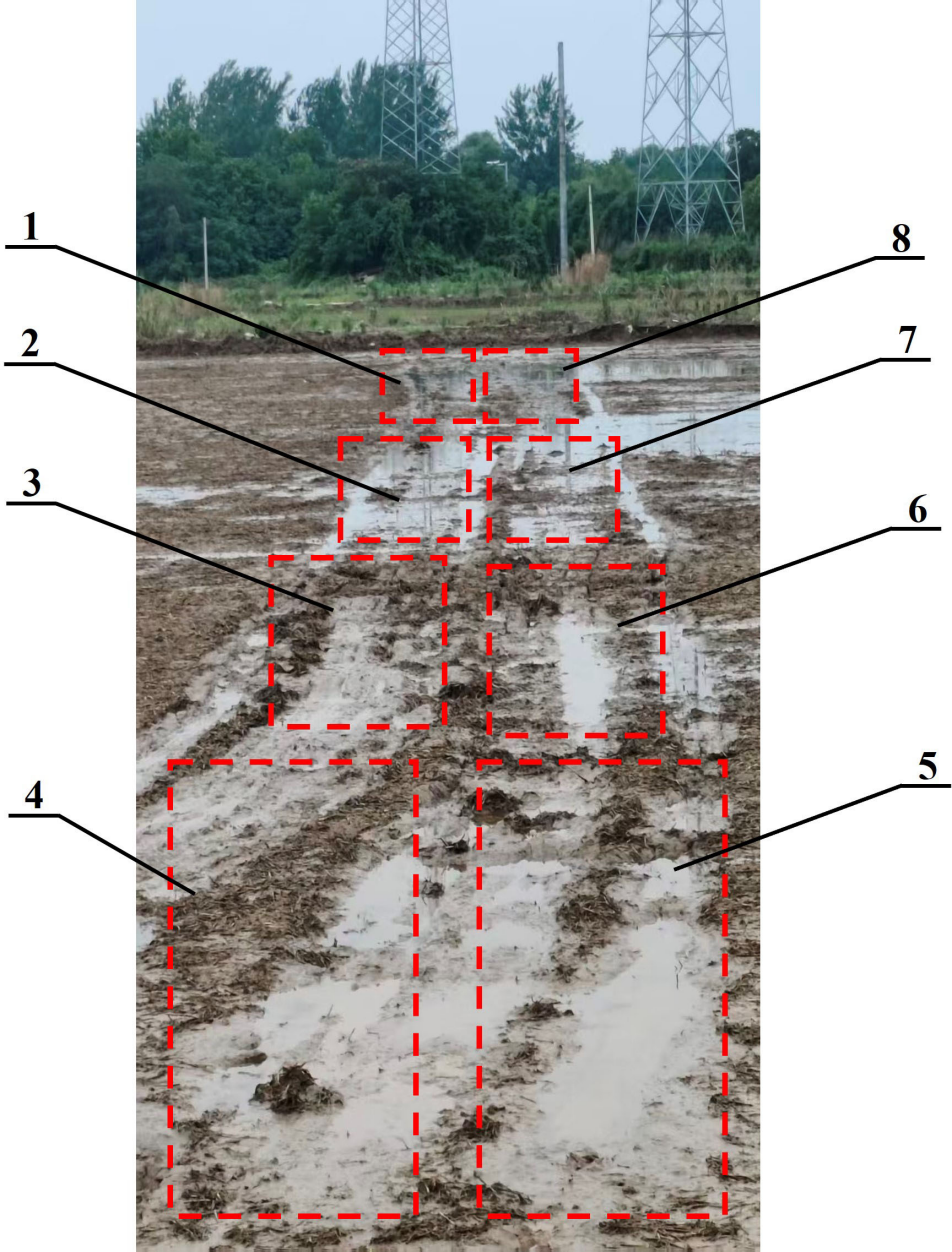
Test site. 1 to 8 are 8 plots.

During the system application test conducted in the experimental field, the vehicle-mounted platform achieved real-time and synchronous monitoring of soil EC_a_, paddy-field temperature, paddy-field moisture, and the tillage-layer depth at each sampling point. Meanwhile, within the test area, soil samples were collected at 2 m intervals using the ring-knife method as reference samples for the EC detection accuracy test. The field experiment was divided into eight standard sampling plots (**Figure 9**). Soil moisture content and soil temperature were measured synchronously in each plot, and the ranges of moisture content and temperature for each plot are listed in **Table 6**.

**Table 6:** Soil moisture and temperature in different experimental plots in the field.

Plot identifier	Soil moisture content (%)	Soil temperature (°C)
1	70.3~71.4	26.6~28.2
2	78.9~82.4	25.1~27.5
3	74.3~76.8	27.3~29.5
4	75.8~78.6	27.5~28.6
5	78.4~80.3	25.6~27.9
6	77.9~80.1	26.2~28.6
7	79.3~83.1	26.3~27.6
8	71.1~73.4	27.8~29.2

**Figure 10:**
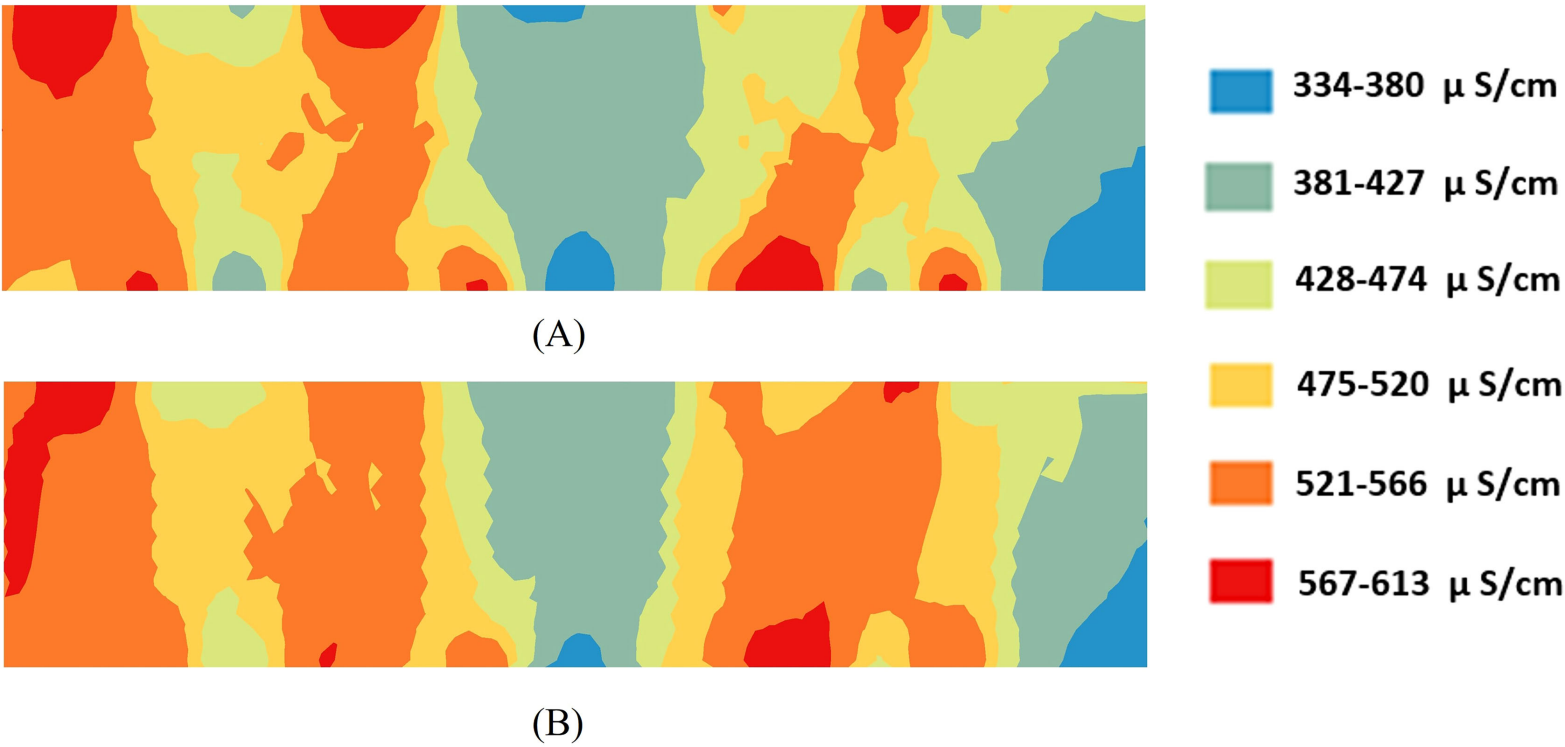
Comparison between vehicle-mounted system EC data and instrument- measured EC data: **(A)** Trend of soil EC in real-time measurements; **(B)** Trend of EC measured by the instrument.

Based on the sensor signals acquired by the vehicle-mounted system, soil EC estimated using the PSO–RBF model was used to generate an interpolated map (**Figure 10A**), while an interpolated map was also produced from soil EC data obtained by the leachate method (**Figure 10B**). The spatial distribution characteristics of the two datasets were further compared using Kriging interpolation. The results indicate that the EC variation trends in **Figures 10A, B** are generally consistent, and both exhibit regular changes with variations in soil temperature and moisture content. In addition, when comparing the reference EC values with the conductivity output from the EC calibration model, the RMSE was 16.3 μS/cm and the mean relative error was 2.70%. These results show that the PSO–RBF-based soil EC model achieved an RMSE of 10.32 μS/cm on static and homogeneous soil samples under laboratory conditions, whereas the RMSE increased slightly under dynamic field conditions with spatial heterogeneity. Nevertheless, the mean relative error remained at approximately 2.70%, indicating that although the calibration accuracy decreases in field environments, it is still within an acceptable range.

### 4.2 Response performance test of the variable fertilization control system

To verify whether the response time of the variable fertilization system controller and the consistency of the fertilization mechanism meet the design requirements, dynamic tests were conducted. The tests included the fertilizer distributor consistency test and the response speed test of the variable fertilization control system. The fertilizer distributor consistency test was performed on an indoor fertilization test bench, while the response speed test of the variable fertilization system was conducted with the system deployed on the rice transplanter.

#### 4.2.1 Fertilizer distributor consistency test

To verify the consistency of the fertilization mechanism in the variable fertilization system, the test was conducted at the Key Laboratory of Agricultural Resource Intelligent Management and Application, Huzhou Normal University. The test was performed on an indoor fertilization test bench, which consisted of a fertilizer box, air-assisted fertilizer delivery device, fertilizer discharge pipe, fertilizer collector, control motor, fertilization controller, and a 12 V battery. The fertilizer used was granular compound potassium sulfate produced by Henan Xinlianxin Chemical Industry Group Co., Ltd., which was dry, uniform in granule size, and free from caking or deliquescence.

The control motor speeds were set at 500, 1000, 1500, 2000, and 2500 r/min, with a working duration of 30 s. The weight of fertilizer collected in each collector was measured using an electronic scale with an accuracy of 0.01 g. Each speed was tested three times, and the mean value was taken as the actual fertilizer discharge at that speed. The test results are shown in **Table 7**.

**Table 7:** The results of the consistency test of fertilizer discharge in each row.

Rotation speed (r/min)	Fertilizer discharge per outlet(g)						Mean(g)	Standard deviation (g)	Coefficient of variation (%)
	No. 1	No. 2	No. 3	No. 4	No. 5	No. 6			
500	334.53	312.48	332.12	329.35	320.45	301.62	321.76	12.82	3.98
1000	667.73	625.82	637.34	650.13	634.86	608.86	637.46	20.18	3.17
1500	940.56	912.92	938.44	978.65	925.13	899.76	932.58	27.35	2.93
2000	1205.16	1169.64	1225.39	1218.42	1181.95	1139.45	1190.00	32.63	2.74
2500	1460.49	1375.37	1420.92	1417.61	1423.34	1359.76	1409.58	36.40	2.58

As shown in **Table 7**, using this system, the maximum coefficient of variation in fertilizer discharge among rows was 3.98%, and the average coefficient of variation was 3.08%, both of which are lower than the 13.00% limit specified in the “Test Methods for Fertilizer Machinery” (GB/T 20346.1—2006) (Huai et al., 2015). This indicates that the fertilizer discharge across all rows is uniform and stable.

#### 4.2.2 Variable fertilizer control system response speed test

To verify the response speed and accuracy of the variable fertilizer system, a response test was conducted at the Qinglu Smart Farm in Danyang, Zhenjiang, Jiangsu Province. The test used a Kubota ride-on rice transplanter as the test platform, with a 12 V onboard battery as the power supply. A high-precision GPS monitoring system was deployed on the transplanter, and collection belts were arranged between the drive wheels.

**Figure 11:**
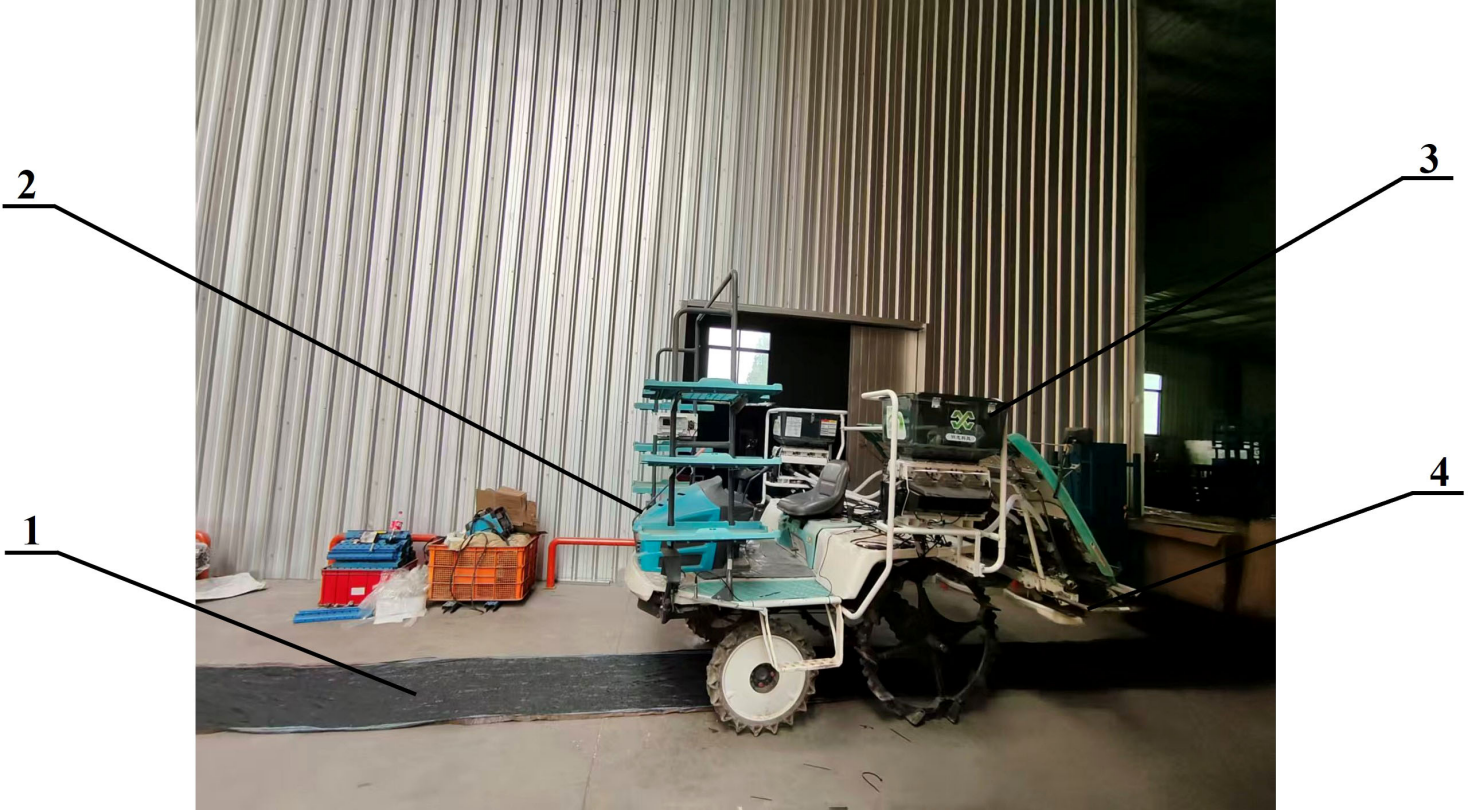
System response speed test. 1. Fertilizer collection belt; 2. SPV-8C25 rice transplanter; 3. Fertilizer device; 4. Fertilizer distributor.

During the test, the transplanter moved at a constant speed of 1 m/s. The soil EC input signal ranged from 300 μS/cm to 600 μS/cm, increasing in increments of 75 μS/cm. To ensure the transplanter reached the target speed when entering the collection belt area, the start position was set 10 m before the beginning of the belt. At the end of the test, the fertilizer collected in each 20 cm segment of the belt was weighed using an electronic balance, and the data were recorded. The test site is shown in **Figure 11**.

**Figure 12:**
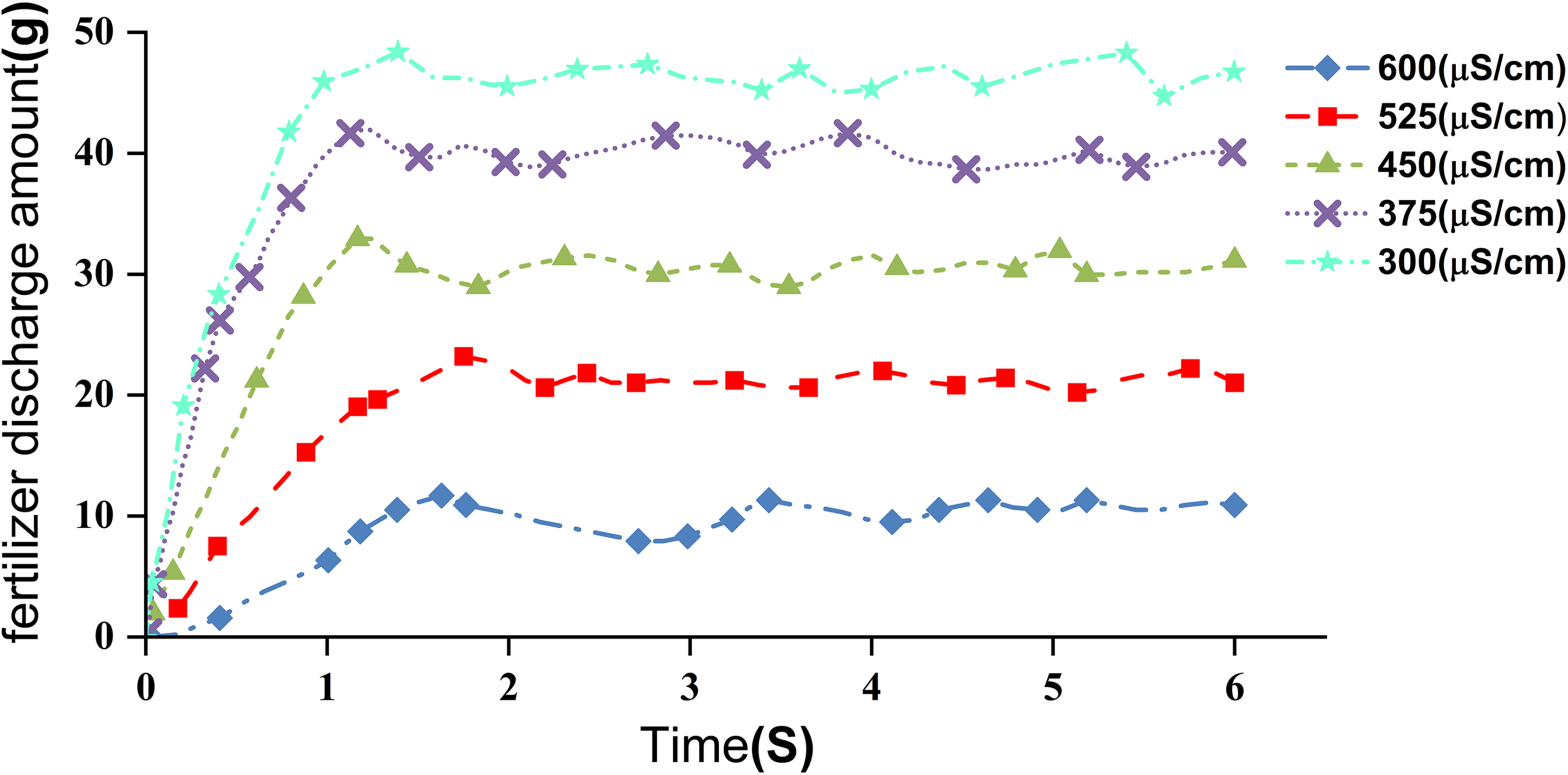
Variable fertilization system response result.

The test results are shown in **Figure 12**. Under the incremental fertilization operation conditions, the system demonstrates ideal dynamic characteristics. The fertilizer application decreases as the conductivity input signal increases, with the error between the actual application amount and the real-time fertilization model calculation results being less than 5%. The maximum response time is 1.60 seconds, and the average response time is 1.28 seconds. Compared with the system proposed by Zhang Jicheng et al (Zhang et al., 2021), the average response time of the system in this study is reduced by approximately 11.7%. This indicates that the system is able to adjust and stabilize the fertilization amount in a short time, meeting the requirements for high responsiveness and accuracy in field operations.

### 4.3 Field trials

**Figure 13:**
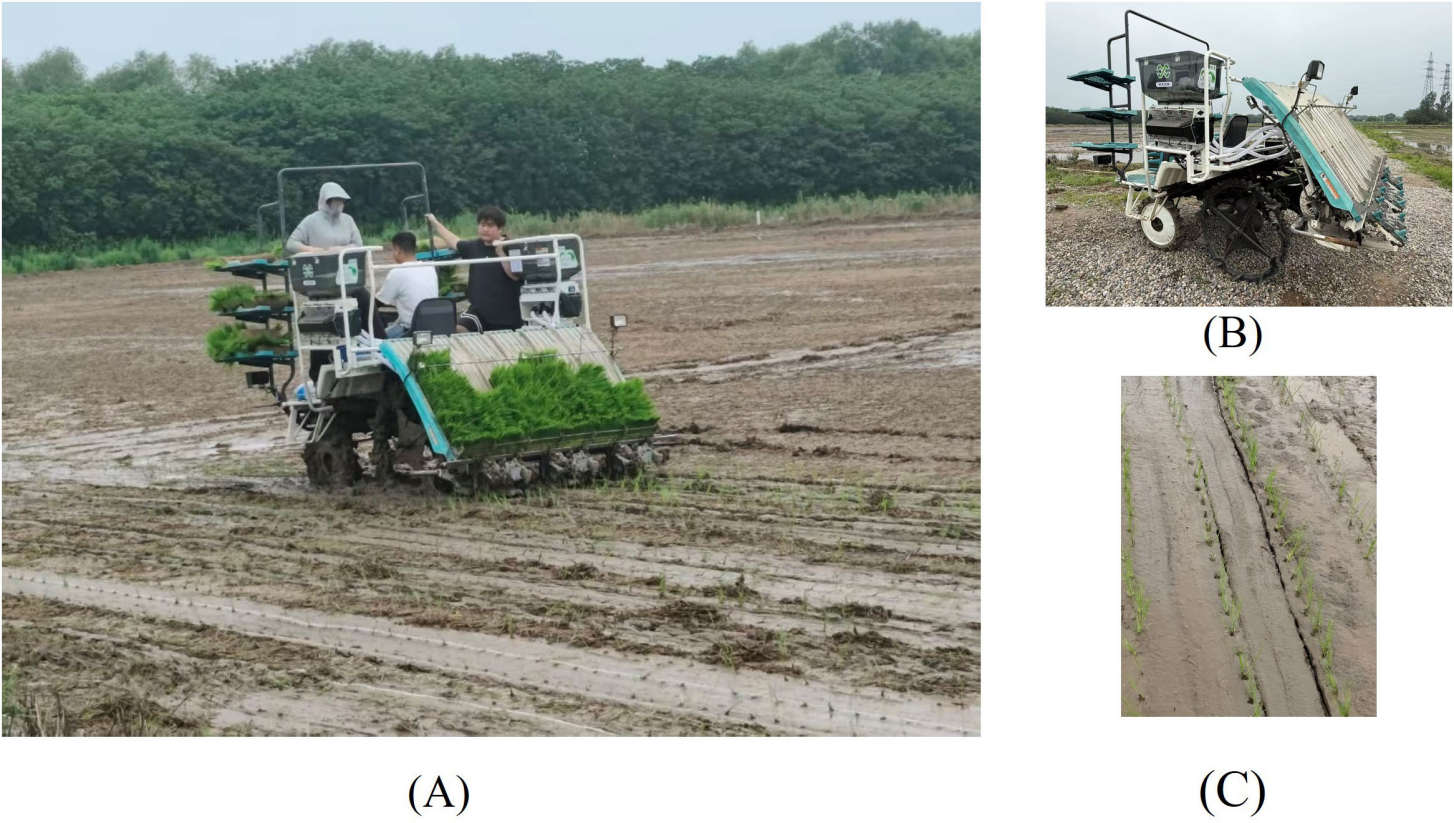
Field test data: **(A)** field experiment; **(B)** Transplanter equipped with variable fertilization system; **(C)** Field test results.

To evaluate the stability of the system during operation and the effectiveness of variable fertilization, field trials were conducted on June 16 and 21, 2025, in experimental plots located in Wuxing District and Nanxun District, Huzhou City, Zhejiang Province. The selected trial areas were 1,400 m² and 1,040 m², respectively. The field trial setup is shown in **Figures 13A–C**.

The trials used controlled-release blended fertilizer (18-7-18) produced by Anhui Maoshi Agricultural Technology Co., Ltd. The rice variety tested was Zhejing 100, with seedlings approximately 20 cm in height during operation.

For the two trial fields, the conventional fertilizer application rates should have been 387 kg/hm² and 450 kg/hm². After applying the variable fertilization system, the actual application rates were 347 kg/hm² and 385 kg/hm², achieving fertilizer reduction rates of 10.34% and 14.44%, with an average reduction rate of 12.39%. The results indicate that the system provides effective fertilizer savings during operation, significantly reducing production costs.

## 5 Discussion

The developed variable side-deep fertilization system for rice integrates multi-sensor data fusion, including dual-electrode EC sensors, ultrasonic sensors, and temperature–humidity sensors, to monitor paddy field conditions in real time. The system corrects the apparent soil EC_a_ through a PSO–RBF-based calibration model and determines the fertilizer application rate based on a fertilization decision model.

A three-factor, four-level full factorial experiment was conducted to investigate the effects of soil moisture content, electrode insertion depth, and soil temperature on soil EC, and a PSO–RBF soil EC correction model was established. The PSO–RBF model was comparatively evaluated against a no-correction approach and a conventional RBF model. The results show that the PSO-optimized RBF model is significantly superior to the no-correction approach and the conventional RBF model in terms of prediction accuracy.

In addition, the system’s incremental PID control algorithm enabled precise adjustment of the control motor speed according to the target fertilizer amount and the transplanter’s travel speed. The response test demonstrated a maximum coefficient of variation of 3.98% for row-wise fertilizer distribution and an average of 3.08%, confirming stable and uniform delivery performance. When the soil EC input ranged from 300 μS/cm to 600 μS/cm, the difference between the actual and model-predicted application rates remained below 5%, with a maximum system response time of 1.60 s and an average of 1.28 s. Compared to similar studies, these results show a significant improvement (Zhang et al., 2021).

These findings verify that the variable fertilization system can accurately respond to real-time soil EC variations and dynamically regulate the fertilizer output, ensuring both precision and stability. Moreover, the soil EC calibration model significantly improved the accuracy of EC correction, with a mean relative error of 2.70%. Collectively, the experimental and field test results demonstrate that the proposed control strategy and sensing system are effective in achieving real-time, adaptive fertilization control for rice production.

## 6 Conclusion

In conclusion, this study developed a variable side-deep fertilization system for rice based on multi-sensor data fusion and a PSO–RBF soil EC calibration model. The system achieved accurate EC correction, reliable control performance, and stable fertilizer delivery. Field validation showed a mean relative error of 2.70% in soil EC correction and an average fertilizer-saving rate of 12.39%. The proposed system provides a theoretical and technical foundation for the mechanization and intelligentization of rice fertilization. It offers a feasible approach for the practical application of soil EC–based variable fertilization and contributes to enhancing fertilizer utilization efficiency and promoting sustainable agricultural development.

## Data Availability

The raw data supporting the conclusions of this article will be made available by the authors, without undue reservation.
